# Cervical lymphatic tissue as an alternative donor site for the microsurgical treatment of secondary lymphedema in limbs^☆^^☆☆^^☆☆☆^

**DOI:** 10.1016/j.bjorl.2025.101600

**Published:** 2025-05-27

**Authors:** Gustavo Amaral de Abreu, Diego Alvarez Naranjo, Felipe de Borba Chiaramonte Silva, Edgar Edinson Fernandez Altamiranda, Sofia Ratchitzki Teixeira, Gabriel Manfro, Gilberto Vaz Teixeira

**Affiliations:** a CEPON, Florianópolis, SC, Brazil; b Universidade Federal de Santa Catarina (UFSC), Florianópolis, SC, Brazil; c São Leopoldo Mandic, Campinas, SP, Brazil; d Hospital Santa Terezinha, Joaçaba, SC, Brazil

**Keywords:** Surgical treatment of lymphedema, Microsurgery, Cervical lymph node dissection

## Abstract

**Objective:**

To describe cervical lymphnodes as an option for a donor area for the transfer of vascularized tissue in lymphedema surgeries.

**Methods:**

Five microsurgical procedures were performed to transfer vascularized lymphatic tissue from cervical stages Ib, III, or IV as the donor site. Patient outcomes were evaluated from 15 to 180 days postoperatively using metric assessments and photographic documentation.

**Results:**

In this study, complete improvement in the measures of certain parts of the affected limb was observed in two of the five patients (100% improvement). Two patients experienced an improvement of over 50%. One patient, lost to follow-up, showed no improvement.

**Conclusion:**

Lymphatic tissue from cervical levels appears to be a viable donor site for the surgical treatment of lymphedema in the limbs. This method may yield promising future outcomes, constituting a new area of practice for head and neck surgeons.

**Level of evidence:**

Level IV.

## Highlights

• Vascularized lymphatic tissue from cervical levels appears to be a viable donor site for the microsurgical transplant treatment of lymphedema.
• In this study, complete improvement in the measures of certain parts of the affected limb was observed in two of the five patients.
• This method may yield promising future outcomes, constituting a new area of practice for head and neck surgeons.

## Introduction

Lymphedema is characterized by the accumulation of lymph in the interstitial tissue. It arises from a dysfunction of the lymphatic system, which is responsible for draining excess fluids and combating microorganisms filtered from the bloodstream. There are numerous risk factors for its development, with malignancies and their treatments being particularly significant.

Tumors may compress lymphatic channels or nodes and can be one of the mechanisms of development. Moreover, the treatment of certain cancers, either surgical or through radiation therapy, often leads to lymphedema as a complication. Its occurrence results in morbidity, worsened quality of life, and increased risk of serious infections in the affected limb. The B-32 trial of the National Surgical Adjuvant Breast and Bowel Project (NSABP) reports lymphedema rates of 8% for breast cancer patients undergoing Sentinel Lymph Node Biopsy and 14% for those undergoing axillary Lymphadenectomy.^1^ In the 1098122023 AMAROS trial of the European Organization for Research and Treatment of Cancer (EORTC), the rates are 13% to 23% for patients after axillary Lymphadenectomy and 5% to 11% for those treated with Radiotherapy.^2^

The diagnosis of lymphedema is predominantly clinical, through volumetric comparison of limbs. Imaging tests can be helpful when there is diagnostic uncertainty even after anamnesis, physical exam, and measurement of the extremities. Lymphoscintigraphy can be particularly useful for diagnosing lymphedema in subclinical phases and is also used for severity classification by some authors.^3^ Based on symptoms and physical examination, lymphedema is classified by severity stage.^4^ This can be done using Foldi’s scale, as outlined in Table 1 (International Society for Lymphology [ISL] stage scale).^5^

**Table 1:** Perimetric data of patients during postoperative follow-up.

Patient Age (years)	Preoperatively	15 days	30‒45 days	60‒90 PO	180 PO	Difference between limbs: metric and percentage			Lymphedema stage	
		Postoperatively	Postoperatively			Pre (%)	PO (%)	% final	Pre	PO
CLS	BD: 37 cm	BD: 36.5 cm	BD: 33 cm	BD: 33 cm	BD: 34 cm	B: 6.5 cm (21%)	B: 3 cm (9%)	54%	B II	B I
Female	AD: 33 cm	AD: 32.5 cm	AD: 32 cm	AD: 33 cm	AD: 30 cm	A: 8.5 cm (34%)	A: 6 cm (25%)	30%	A II	A II
59	BE: 30.5 cm	BE: 31 cm	BE: 31 cm	BE: 31 cm	BE: 31 cm					
EVS	AE: 24.5 cm	AE: 24.5 cm	AE: 24 cm	AE: 24.5 cm	AE: 24 cm	C: 0 cm (0%)	C:2 cm (0%)	0%	C 0	C 0
	CE: 51 cm	CE: 45 cm	CE: 48 cm	CE: 48 cm	CE: 48 cm					
Male	PE: 41.5 cm	PE: 35 cm	PE: 38 cm	PE: 36 cm	PE: 36 cm	P: 3 cm (7.8%)	A: 3 cm (0%)	100%	P I	P 0
55	TE: 32.5 cm	TE: 25 cm	TE: 30 cm	TE: 26 cm	TE: 26 cm	T: 8.5 cm (35%).	T: 2 cm (8.3%)	77%	T II	T I
	CD: 51 cm	CD: 51 cm	CD: 50 cm	CD: 51 cm	CD: 51 cm					
	PD: 38.5 cm	PD: 38.5 cm	PD: 38 cm	PD: 39 cm	PD: 39 cm					
	TD: 24 cm	TD: 24 cm	TD: 24 cm	TD: 24 cm	TD: 24 cm					
JMRS	BD: 31 cm			BD: 32 cm		B: 4 cm (14%)	B: 4 cm (14%)	0%	B I	B I
Female	AD: 31 cm			AD: 30 cm		A: 9 cm (40%)	A: 8 cm (36%)	12%	A III	A II
66	BE: 27 cm			BE: 28 cm						
	AE: 22 cm			AE: 22 cm						
FAS	BD: 41 cm	BD: 37 cm				B: 3 cm (7,8%)	B: -−1 cm (0%)	100%	B I	B 0
Female	AD: 30 cm	AD: 31 cm				A: 0 cm (0%)	A: 1 cm (3%)	0%	A 0	A 0
52	PUD: 25 cm	PUD: 21.5 cm				PU: 1 cm (4%)	A: -−2.5 cm (0%)	100%	M II	M 0
	MD: 25.5 cm	MD: 22.5 cm				M: 4.5 cm (21%)	M: 0.5 cm (2%)	89%		
	BE: 38 cm	BD: 38 cm								
	AE: 30 cm	AD: 30 cm								
	PUE: 24 cm	PU: 24 cm								
	ME: 21 cm	M: 21 cm								
SSC										
Female										
70										

B (arm); A (forearm); P (leg); T (ankle); C (thigh); M (hand); PU (wrist); PO (postoperatively); Pre (preoperatively); D (right); E (left).

The treatment of lymphedema involves general measures such as limb elevation, diet, physical activity, and skin care to prevent injuries that could serve as gateways for infectious agents. For mild lymphedema (stage I of the ISL scale), beyond general measures, physiotherapy and compression garments can be beneficial. For patients classified as stage II and III in the ISL scale, indicating moderate to severe lymphedema, physiotherapy and compression are more intensively applied, yet not as effective as surgical treatment. A randomized study compared conservative treatment with surgical treatment and observed a significant advantage for the latter (57% volume reduction vs. 18% for conservative treatment), along with symptom improvement and fewer infectious episodes.^6^ Hence, failure of conservative treatment calls for an evaluation of the available surgical options. Similarly, recurrent cellulitis, primary malformations, severe symptoms, functional limitation, deformities, and patient suffering indicate the need for surgical intervention. As described by Corinne Becker,^7^ vascularized lymph node transfer is an excellent surgical management option for lymphedema, providing permanent volume reduction of the affected limb, reducing infection risk, and enhancing patient quality of life.

This study presents a technique based on vascular anastomosis, both arterial and venous, using donor lymphatic tissue from a site proximal to the affected area. The most common primary donor sites include the submental, inguinal, supraclavicular, and omental areas, and the lateral thoracic chain. The cervical region offers suitable tissue with minimal risk of secondary edema in the donor site.^8^, ^9^ Efforts to treat this severe morbidity should be encouraged to minimize suffering and improve the quality of life of patients.^10^

This study aimed to present the dissection of cervical lymph node stages as an option for donor sites for the transfer of vascularized tissue in lymphedema surgery.

## Methods

This study was conducted at the Center for Oncological Teaching and Research (CEPON), which is a reference for cancer treatment in the state of Santa Catarina Brazil. Candidates for surgery were selected between August 2022 and June 2023.

Selection criteria included being at least 18-years-old, having undergone cancer treatment, having the base disease under oncological control, presenting stage 0 or 1 in the Eastern Cooperative Oncology Group (ECOG) performance status scale, and suffering from stage I, II, or III lymphedema (ISL stage scale).

**Fig. 1:**
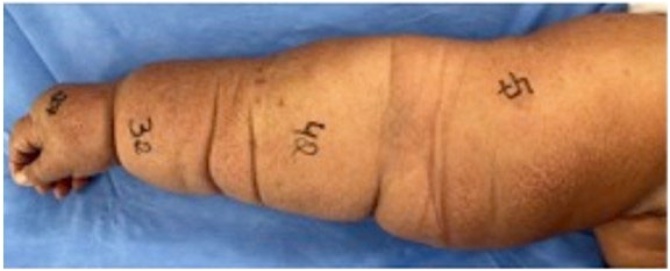
Right upper limb of patient SSC with respective preoperative measures for arm, forearm, wrist, and hand.

The diagnosis of lymphedema was based on clinical examination, and measures of the circumference of the affected limb, as well as the contralateral limb for comparative purposes, were taken at the first consultation (Fig. 1).

**Fig. 2:**
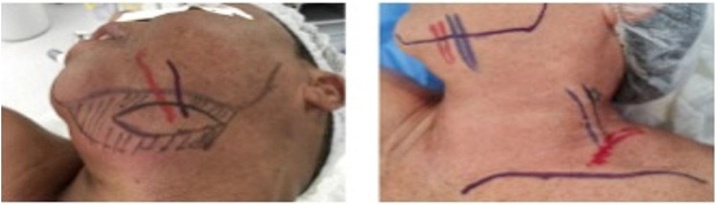
Patients CLS and SSC with schematic markings of the cervical donor sites highlighting the main arteriovenous pedicle.

Patients were then subjected to vascularized lymph node transfer surgery from cervical stages Ib, III, or IV as the donor site (Fig. 2).

**Fig. 3:**
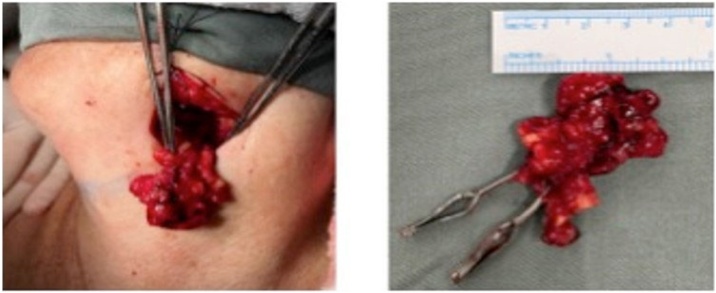
Intraoperative view of patient FAS showing the dissected lymphatic tissue corresponing to cervical level Ib with a detailed view after removal.

Dissection of the lymphatic tissue from the chosen donor site was performed preserving its arteriovenous pedicle (Fig. 3).

In the recipient area, dissection of an artery and a vein of compatible calibers was performed, as well as lysis of fibrosis.

**Fig. 4:**
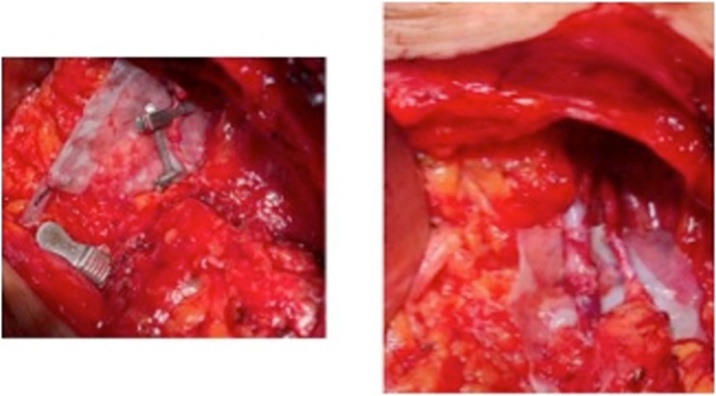
Detail of arteriovenous anastomosis for patient SSC during surgery.

Arteriovenous anastomosis was then carried out using microsurgical instruments, 6× magnification lenses, and 8.0 nylon (Fig. 4).

In the case of a patient suffering from stage III lymphedema, because of the severe deformation of the limb, resection of excess tissue and release of areas of skin constriction were added with flap rotation techniques.

None of the patients presented complications in the immediate postoperative period. Four of them were discharged on the first postoperative day. The patient with stage III lymphedema who also underwent brachioplasty during surgery was discharged on the fifth postoperative day. After hospital discharge, the patients were referred back to physical therapy services.

Still on an outpatient basis, the patients were evaluated by the medical team on the 15^th^ day, between the 30^th^ and the 45^th^ day, between the 60^th^ and the 90^th^ day, and after 180 days, postoperatively. During the consultations, limb perimetry was measured for comparative purposes, and some results were photographically documented.

## Results

Five patients were selected, four females aged 52 to 70 years with lymphedema in the Right Upper Limb (RUL), and one male aged 55-years whose lymphedema affected the Left Lower Limb (LLL). The four female patients had a history of treatment for breast cancer, and the male patient had myxoid liposarcoma in the left thigh. All of them underwent oncological treatment with combined chemotherapy and radiotherapy, and surgery.

The preoperative and postoperative measures of each patient are shown in Table 1, as well as the classification of each one’s lymphedema according to the ISL stage scale.

**Fig. 5:**
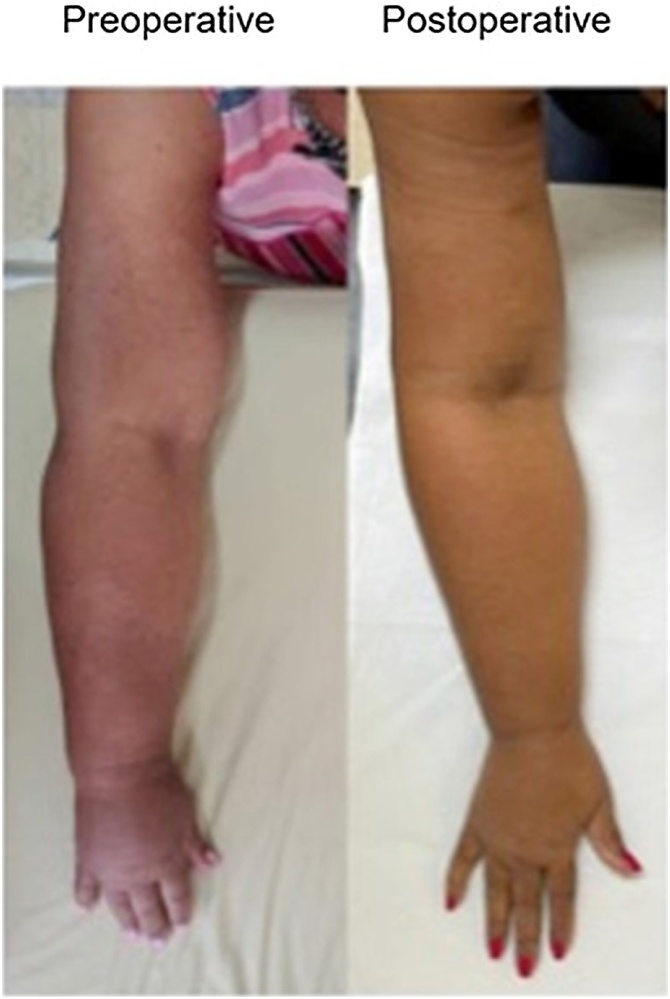
Comparative photo of patient CLS preoperatively and at 180-days postoperatively.

The first patient, CLS, a 59-year-old female with lymphedema in the RUL, had a preoperative metric difference between the right and left arm of 6.5 cm (which corresponds to a 21% increase in the affected limb), classifying her as having stage II Lymphedema (ISL). For the preoperative forearm measure, she had an 8.5 cm difference (34% increase ‒ ISL stage II). After 180 days of postoperative follow-up, the metric difference for the arm was 3 cm and 6 cm for the forearm (9% and 25% relative increase of the affected limb – ISL stage I for the arm and stage II for the forearm). Thus, the arm improved as the difference in measure between limbs decreased from 6.5 cm to 3 cm (a reduction of 54%) (Fig. 5).

The second patient, EVS, a 55-year-old male with lymphedema in the LLL, had no preoperative difference between the left and right thigh. For the leg, the difference was 3 cm (7.8% increase – ISL stage I), and for the ankle, a difference of 8.5 cm (35% increase – ISL stage II). The left thigh measure at 180-days postoperatively was smaller than that of the right thigh (48 and 51 cm, respectively), resulting in −3 cm (ISL stage 0). For the ankle, the difference at 180 days postoperatively was 2 cm (8.5% comparative increase), moving from ISL stage II to stage I.

The third patient, JMRS, a 66-year-old female, had a difference between the affected RUL and the healthy left upper limb of 4 cm for the arm (14% relative increase) and 9 cm for the forearm (40% relative increase). The preoperative classification for the arm was ISL stage I and for the forearm, ISL stage III. These differences remained at the postoperative consultations of 60 and 90 days for the arm, and for the forearm, there was only a 1 cm relative decrease (from 9 cm to 8 cm).

The fourth patient, FAS, a 52-year-old female with lymphedema in the RUL, had a preoperative measure difference between the arms of 3 cm (7.8% relative increase). There was no preoperative difference for the forearm of this patient; a difference of 1 cm for the wrist (4% relative increase) and 4.5 cm for the hand (21% relative increase). In the first 15-days postoperatively, the difference between the arm measures was −1 cm (the measure of the right arm minus the measure of the left arm); for the forearm, this difference was 1 cm (3%); for the wrist, it was −2.5 cm, and for the hand, 1.5 cm (7% relative increase). Her final ISL classification was reduced from stage I to 0 for the arm and from stage II to 0 for the hand.

**Fig. 6:**
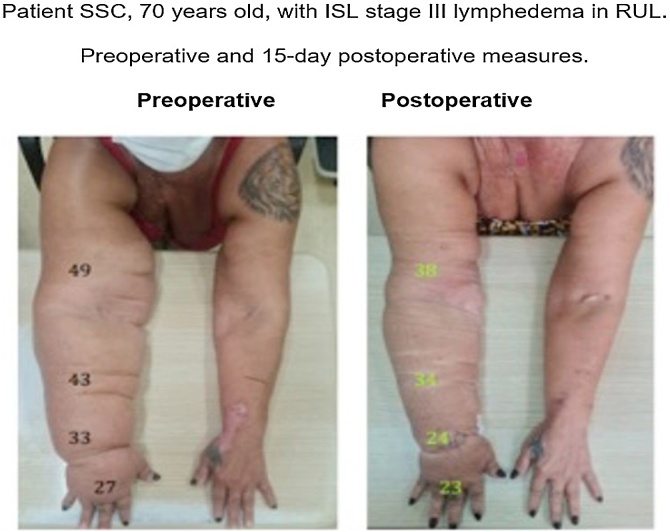
Comparative photo of patient SSC preoperatively and at 15 days postoperatively. Detail of the measures of the arm, forearm, wrist, and hand.

Finally, the fifth patient, SSC, a 70-year-old female with stage III ISL lymphedema of the RUL, had a difference between arm measures of 17 cm (53% relative increase); 20 cm for the forearm (87% relative increase); 16 cm for the wrist (94% relative increase), and 6 cm for the hand (28% relative increase). Fifteen days postoperatively, these measures were 8 cm for the arm (26% relative increase), 11 cm for the forearm (47% relative increase), 7 cm for the wrist (41% relative increase), and 3 cm for the hand (15% relative increase). Thus, she reduced her ISL classification from stage III to II for the arm and from stage II to I for the hand (Fig. 6).

## Discussion

The type of cancer most commonly associated with lymphedema is breast cancer. Bergmann et al.,^11^ in a literature review, reported a prevalence of lymphedema in patients undergoing axillary lymphadenectomy ranging from 6% to 49%, and an incidence of 0% to 22%.

In a meta-analysis of lymphedema secondary to cancer, Cormier et al.^12^ referred to an overall incidence rate of 15.5%, and according to the type of malignancy, the rate for sarcoma was 30%. Following this trend, of the five patients selected for the study, four had a history of treatment for breast cancer with surgery, including lymph node dissection and radiotherapy. Only one had liposarcoma.

**Fig. 7:**
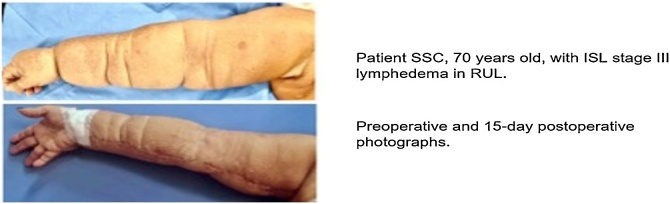
Comparative photo of patient SSC preoperatively and at 15-days postoperatively. Focus on the healing of the arm and forearm, as well as the reduction in volume observed postoperatively.

All had experienced failure with conservative treatment. Two of them (CLS and EVS) also had chronic pain and previous episodes of infection in the affected limb. One patient, SSC, had stage III lymphedema with severe limb deformity. Becker et al.^7^ described long-term improvement rates of over 90% for patients undergoing vascularized lymph node transplant as a surgical treatment for lymphedema (41% of patients achieving cure and 50% downstaging). Similarly, in this study, two patients showed a 100% improvement for at least one limb measure (EVS and FAS) and another two showed an improvement of over 50% (CLS and SSC). One of the patients (JMRS), lost to follow-up, showed no improvement with the treatment. The treatment of stage III lymphedema involves a surgical combination of physiological techniques, such as the transfer of lymphatic tissue, and reductive techniques. One of the patients in the study (SSC), who had the highest stage of lymphedema, underwent this combined technique. For most measures, she obtained improvement rates of over 50% (Fig. 7).

The choice of treatment for lower limb lymphedema in patient EVS may raise questions about whether the small volume of cervical lymph nodes can provide adequate drainage for the lower limb region. Cheng et al.^13^ reported an average of 3.3 lymph nodes dissected in the submental region. This was the donor site chosen by these authors for the treatment of lower limb lymphedema with satisfactory results. As an average reduction rate in circumference, they referred values of 64 ± 1.5% for measures above the knee; 63.7 ± 34.3% below the knee, and 67.3 ± 19.2% above the ankle. For patient EVS, the results were equally significant, with normal values for the leg at 30- and 45-days postoperatively and a 77% improvement for the ankle at 180-days postoperatively. The good response to treatment also extended to symptomatic improvement and the infectious episodes presented by this patient. Similar to the first patient, this one also underwent treatment for chronic pain and, like the other, had previously experienced erysipelas in the affected limb on other occasions.

In a randomized study, Dimitrios Dionyssiou et al.^6^ described an improvement in episodes of skin infection, in addition to the esthetic and functional outcome for patients undergoing lymphedema treatment.

**Fig. 8:**
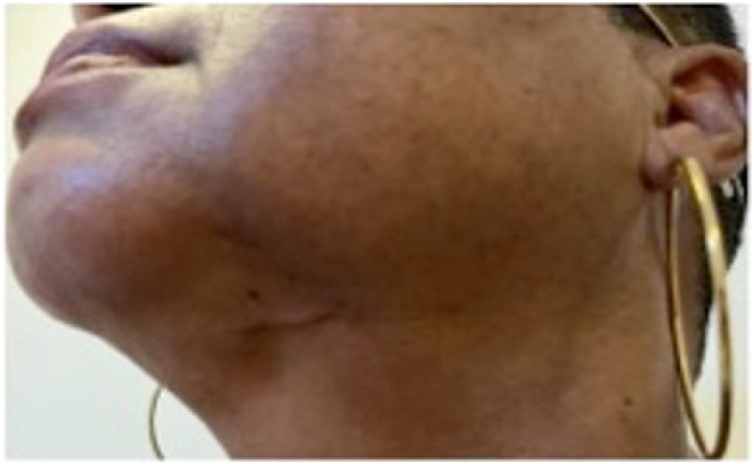
Healing of the donor site of patient CLS in the late postoperative period.

All patients showed a reduction in the stage of lymphedema for at least one comparative measure between limbs. None of the patients had postoperative complications (Fig. 8, representing the healing of the cervical donor site in the late postoperative period for patient CLS).

## Conclusion

Lymphatic tissue from the cervical levels appears to be a viable donor site for the surgical treatment of lymphedema, yielding promising future outcomes, and offering versatility by providing effective tissue to treat both upper and lower limb lymphedema. It also has applicability as a technique in the treatment of stage III lymphedema. This method constitutes a new area of practice for head and neck surgeons.

## Funding

None.

## Declaration of competing interest

The authors declare no conflicts of interest.
